# Perturbations of Monocyte Subsets and Their Association with T Helper Cell Differentiation in Acute and Chronic HIV-1-Infected Patients

**DOI:** 10.3389/fimmu.2017.00272

**Published:** 2017-03-13

**Authors:** Peng Chen, Bin Su, Tong Zhang, Xiaojing Zhu, Wei Xia, Yan Fu, Guoxian Zhao, Huan Xia, Lili Dai, Lijun Sun, Lifeng Liu, Hao Wu

**Affiliations:** ^1^Center for Infectious Diseases, Beijing You’an Hospital, Capital Medical University, Beijing, China; ^2^Beijing Key Laboratory for HIV/AIDS Research, Beijing, China

**Keywords:** acute HIV-1 infection, monocyte subsets, CD163, immune activation, T helper cells

## Abstract

Monocytes have been recently subdivided into three subsets: classical (CD14++CD16−), intermediate (CD14++CD16+), and non-classical (CD14+CD16++) subsets, but phenotypic and functional abnormalities of the three monocyte subsets in HIV-1 infection have not been fully characterized, especially in acute HIV-1 infection (AHI). In the study, we explored the dynamic changes of monocyte subsets and their surface markers, and the association between monocyte subsets and the IFN-γ, interleukin (IL)-4, IL-17, and TNF-α producing CD4+ T cells in acute and chronic HIV-1-infected patients. We found that, in the acute HIV-1-infected individuals, the frequency of the intermediate CD14++CD16+ monocyte subsets, the CD163 density and HLA-DR density on intermediate CD14++CD16+ monocytes, and plasma soluble form of CD163 (sCD163) were significantly higher than that in healthy controls. Intermediate CD14++CD16+ monocyte subsets and their HLA-DR expression levels were inversely correlated with the CD4+ T cell counts, and the intermediate CD14++CD16+ monocytes were positively correlated with plasma sCD163. In contrast to the non-classical CD14+CD16++ and classical CD14++CD16− monocyte subsets, the frequency of the intermediate CD14++CD16+ monocytes was positively associated with the frequency of IFN-γ and IL-4 producing CD4+ T cells in HIV-1-infected patients. Taken together, our observations provide new insight into the roles of the monocyte subsets in HIV pathogenesis, particularly during AHI, and our findings may be helpful for the treatment of HIV-related immune activation.

## Introduction

The interactions between the virus and the immune system during acute HIV-1 infection (AHI) determine the viral load set point and other critical events. Monocytes can act as regulators of the immune system, and monocytes abnormalities are responsible for the hyperactivity observed in HIV-1-infected patients (1). In 2010, three monocyte subsets were recommended by the Nomenclature Committee of the International Union of Immunological Societies: classical (CD14++CD16−), intermediate (CD14++CD16+), and non-classical (CD14+CD16++) subsets (2). Phenotypic and functional abnormalities of the three monocyte subsets have not been fully characterized in HIV-1 infection, especially in acute HIV-1 infection.

Identification of the three subsets within monocytes populations has put monocyte heterogeneity into sharp focus. The perturbation of the three monocyte subsets has been examined in various diseases including bacterial infection, viral infection, and autoimmune disease (3–5). Analysis of monocyte subsets may provide useful clinical parameters in various settings (6). In chronic HIV-1-infected patients, both the intermediate and non-classical subsets were increased, and intermediate subsets were inversely correlated with a decrease in CD4+ T-cell counts (7). In response to the exogenous and endogenous stimulations, monocytes express a variety of surface markers. CD163 is expressed exclusively on monocytes and macrophages, during activation, CD163 can be shed as soluble form of CD163 (sCD163) (8). It was found that CD163 expression levels on intermediate CD14++CD16+ monocytes of chronic HIV-1-infected subjects were significantly higher than that of healthy controls (HC) (9). Plasma sCD163 was evaluated as an independent marker of the progression to death and AIDS (10). In chronic HIV-1-infected individuals, there was a higher density of HLA-DR on monocytes, and combined antiretroviral therapy (cART) has decreased the density of HLA-DR on inflammatory monocytes (CD14+CD16+) (11). Until now, the perturbations of the three monocyte subsets and their surface markers have remained incompletely understood, especially in acute HIV-1-infected individuals.

Depending on the cytokine environment, naïve CD4+ T cells differentiate into T helper (Th) 1, Th2, Th17, and other lineages, each lineage has distinct biological functions. In immune thrombocytopenia, CD16+ monocytes promoted Th1 development, which in turn negatively regulated interleukin (IL)-17 and Treg induction (12). In rheumatoid arthritis, the CD14brightCD16 monocyte subsets promoted an expansion of the Th17 cell population (13). The specific roles of the three monocyte subsets on Th cell differentiation have not been fully characterized in HIV-1-infected patients.

From 2007 to 2012, 484 acute HIV-1-infected cases among 5,800 men who have sex with men (MSM) were identified by our team (14). We found that acute HIV-1 infection improved the immune responses against chronic hepatitis B (15). In the present study, we explored dynamic changes of the monocyte subsets and their surface markers, and the association between monocyte subsets and the IFN-γ, IL-4, IL-17, and TNF-α producing CD4+ T cells in acute and chronic HIV-1-infected patients.

## Materials and Methods

### Patients

All the participants provided written informed consent for their information, and clinical samples were stored and used for research. This study and all relevant experiments have been approved by the Beijing You’an Hospital Research Ethics Committee and informed consent was provided according to the declaration of Helsinki. The methods were carried out in accordance with approved guidelines and regulations.

Thirty-seven homosexual men with acute HIV-1 infection (AHI) were enrolled in the study, these patients were recruited from an HIV-1-negative high risk MSM cohort who were screened every 3 months for HIV-1 infection at Beijing You’an Hospital. The progression of early HIV-1 infection can be depicted as six discrete stages as proposed by Fiebig et al. (16). These acute HIV-1-infected patients were at Fiebig stage III-V. Thirty-one chronic HIV-1-infected cART-naïve patients (CHI&ART−) and 63 chronic HIV-1-infected patients on cART (CHI&ART+) were randomly enrolled from the HIV/AIDS clinic of Beijing You’an Hospital. All HIV-1-infected patients in the study were MSM without HBV/HCV coinfection and other comorbidities, none of them were drug users. Chronic HIV-1-infected patients were diagnosed at least 1.2 years (median 1.8 years, range from 1.2 to 2.6 years) before enrollment. Sixty-three chronic HIV-1-infected patients in the study were treated with the following regimen: TDF+3TC+EFV, which was recommended as a first-line regimen in China. Sixty-three patients in the study have been on treatment for more than 1 year, and the viral loads of these patients are undetectable. Forty-two male homosexual HC were also included in the study. The ages of all groups were matched.

### Cell Surface and Intracellular Cytokine Staining

Cryopreserved peripheral blood mononuclear cells (PBMCs) were used, and cell viability was evaluated in cell surface and intracellular cytokine staining experiment. Cryopreserved PBMCs were thawed in RPMI 1640 medium (Invitrogen, Carlsbad, CA, USA), washed with PBS containing 1% BSA, and then incubated at room temperature for 20 min with the cells viability marker fixable viability stain 510 (BD Biosciences, San Jose, CA, USA).

Monocyte phenotypic analysis was performed after staining with anti-CD14-FITC (eBioscience), anti-CD16-PE (eBioscience), anti-HLA-DR-PerCP-Cyanine5.5 (eBioscience), and anti-CD163-APC (eBioscience Inc., San Diego, CA, USA). Intracellular IFN-γ, IL-4, IL-17, and TNF-α staining was performed after stimulating with a leukocyte activation cocktail with BD GolgiPlug (BD Bioscience, San Jose, CA, USA) for 5 h, the leukocyte activation cocktail contained PMA, ionomycin, and brefeldin A. Cells were initially stained with anti-CD4-PE-Cy7 (eBioscience Inc., San Diego, CA, USA), cells were then fixed and permeablized with Cytofix/Cytoperm according to the manufacturer’s instructions (BD Biosciences, San Jose, CA, USA), followed by staining with anti-IL-4-PE (eBioscience), anti-IL-17-APC (eBioscience), anti-IFN-γ-eFluor 450 (eBioscience), anti-TNF-α-PerCP-Cyanine5.5 (eBioscience Inc., San Diego, CA, USA).

All expression analyses were performed by flow cytometry using BD FACSCanto™ II with Diva software (BD Biosciences, San Jose, CA, USA). Forward scatter and side scatter light gating were used to exclude cell debris from the analysis. Forward height and forward area were used to exclude doublet cells. The final analysis was performed using the Flowjo 10.0.7 software (Tree Star Inc., Ashland, OR, USA).

### CD4+ T-Cell Count and Viral Load Measurement

The CD4+ T-cell count was determined by using anti-CD3-APC, anti-CD4-FITC, and anti-CD8-PE monoclonal antibodies (BD Biosciences). Analysis was then carried out using a BD FACSCanto™ II Flow cytometry (BD Biosciences, San Jose, CA, USA) system. HIV-1 viral load tests were done by using an automated real-time PCR-based *m*2000 system (Abbott Molecular Inc., Des Plaines, IL, USA) according to manufacturers’ instruction, and the sensitivity of detection was 40 copies per milliliter.

### Quantification of Soluble CD163 by Using ELISA

Quantification of soluble CD163 (sCD163) was determined by using a commercial ELISA Kit (R&D Systems, Inc., USA) according to the manufacturer’s instruction without modification.

### Statistical Analysis

Statistical analysis was performed by using an ANOVA test, Student’s *t*-test, or non-parametric tests. All reported *p* values were two sided and considered significant at *p < 0.05 or **p < 0.01. The association was evaluated by Spearman’s correlation test. All data were analyzed using SPSS 21.0 statistical software (Chicago, IL, USA).

## Results

### Characteristics of Participants

Thirty-seven homosexual men with acute HIV-1 infection (AHI), 31 CHI&ART-, 63 chronic HIV-1-infected patients with undetectable viral load after cART (CHI&ART+), and 42 HC were enrolled in the study. The information of numbers, ages, viral loads, cell counts, and CD4+/CD8+ ratio is presented in Table 1. The age and sex of the HIV-1-infected patients and health controls are matched, and the viral loads of the health controls are not available. The mean CD4 counts and CD4/CD8 ratio of HC are higher than those of the acute HIV-1-infected patients (*p* < 0.01) and higher than those of chronic HIV-1-infected patients without cART (*p* < 0.01).

**Table 1:** **Characteristics of HIV-1-infected patients and healthy controls**.

	Healthy control	Acute HIV-1-infected patients	Chronic cART-naïve HIV-1-infected patients	Chronic HIV-1-infected patients with cART
Cases, no	42	37	31	63
Age (years)	29.6 (20–50)	31.4 (22–49)	30.3 (20–52)	32.8 (21–54)
HIV-RNA (copies/ml)	NA	666,265.6 (3,685–5,536,452)	26,347.3 (567–147,548)	TND
CD4 (cells/μl)	716 (427–1,322)	353 (137–497)	447 (238–756)	562 (176–897)
CD4/CD8 ratio	1.58 (0.63–2.59)	0.35 (0.12–1.58)	0.64 (0.24–1.62)	1.07 (0.33–2.36)

*Data are presented as the means with ranges*.

*cART, combined antiretroviral therapy; NA, not available; TND, target not detected*.

### Perturbations of the Three Monocyte Subsets in HIV-1-Infected Individuals

**Figure 1:**
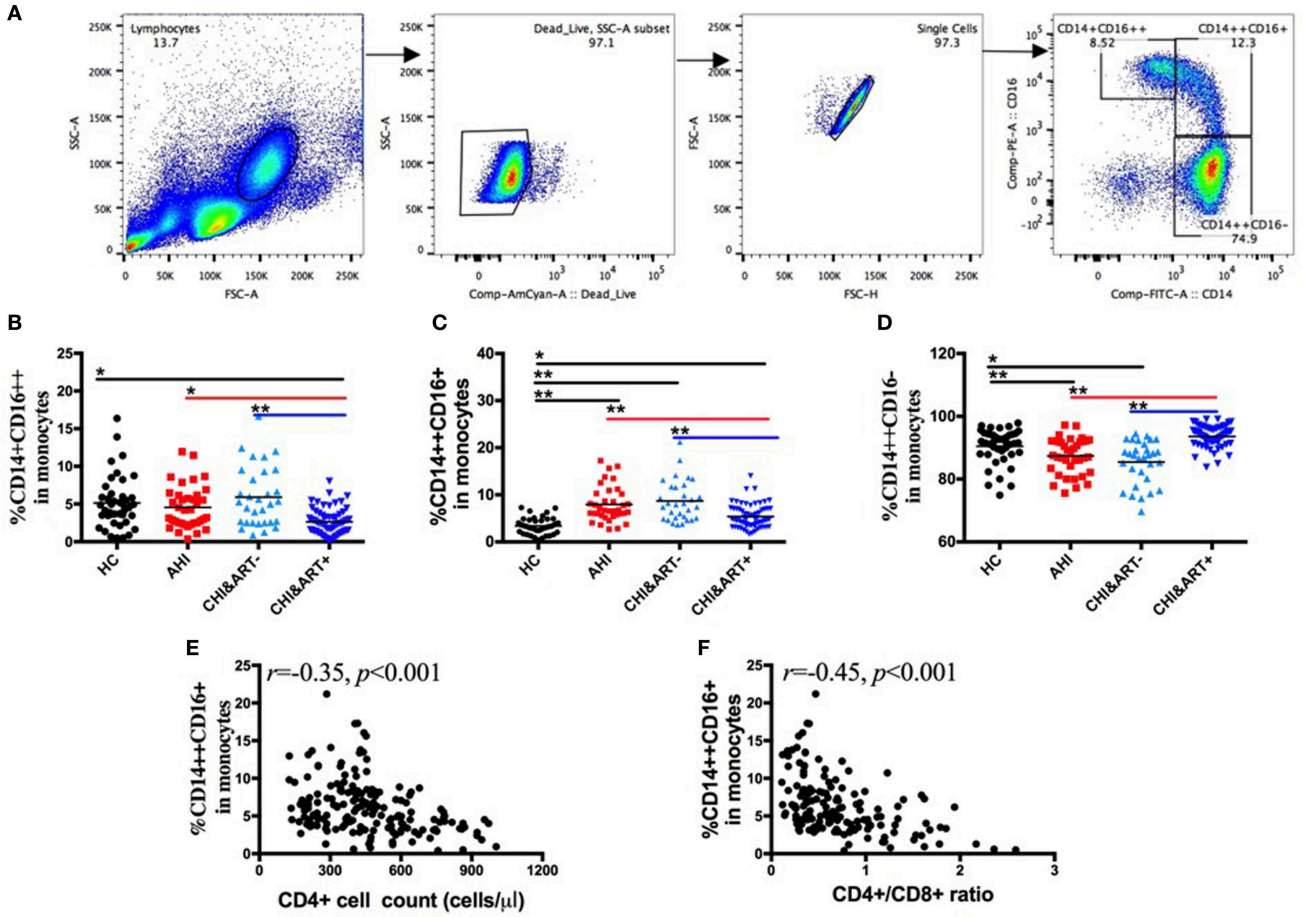
**Perturbations of the three monocyte subsets in HIV-1-infected individuals**. **(A)** The gating strategy for analysis of classical CD14++CD16−, intermediate CD14++CD16+, and non-classical CD14+CD16++ monocyte subsets is indicated. Frequencies of CD14+CD16++ **(B)**, CD14++CD16+ **(C)**, and CD14++CD16− **(D)** monocytes were analyzed by flow cytometry in healthy controls (HC), the patients with acute HIV-1 infection (AHI), chronic HIV-1-infected cART-naïve patients (CHI&ART-), and chronic HIV-1-infected patients with undetectable viral load after cART (CHI&ART+). All *p* values were calculated using an ANOVA, Student’s *t*-test, or the Mann–Whitney *U* test, *p < 0.05, **p < 0.01. Correlations between the frequency of the CD14++CD16+ monocytes and CD4 T cell counts **(E)** as well as the CD4/CD8 ratio **(F)** calculated by the Spearman correlation test (HC, AHI, CHI&ART−, and CHI&ART+ groups).

Based on CD14 and CD16 expression, monocytes are divided into three subsets: classical (CD14++CD16−), intermediate (CD14++CD16+), and non-classical (CD14+CD16++) subsets. The gating strategy is shown in Figure 1A. As seen in Figure 1, compared with HC, intermediate CD14++CD16+ monocytes (Figure 1C) are increased in both AHI and CHI&ART− patients, whereas classical CD14++CD16− monocytes are decreased in AHI and CHI&ART− patients (Figure 1D). Although cART partially recovered the proportions of the three monocyte subsets, monocytes perturbations in HIV-1-infected individuals still existed after cART (Figures 1B,C). The intermediate CD14++CD16+ monocytes are inversely correlated with CD4 cell counts (Figure 1E) and the CD4/CD8 ratio (Figure 1F).

### Perturbations of Monocyte Phenotypes in HIV-1-Infected Individuals

**Figure 2:**
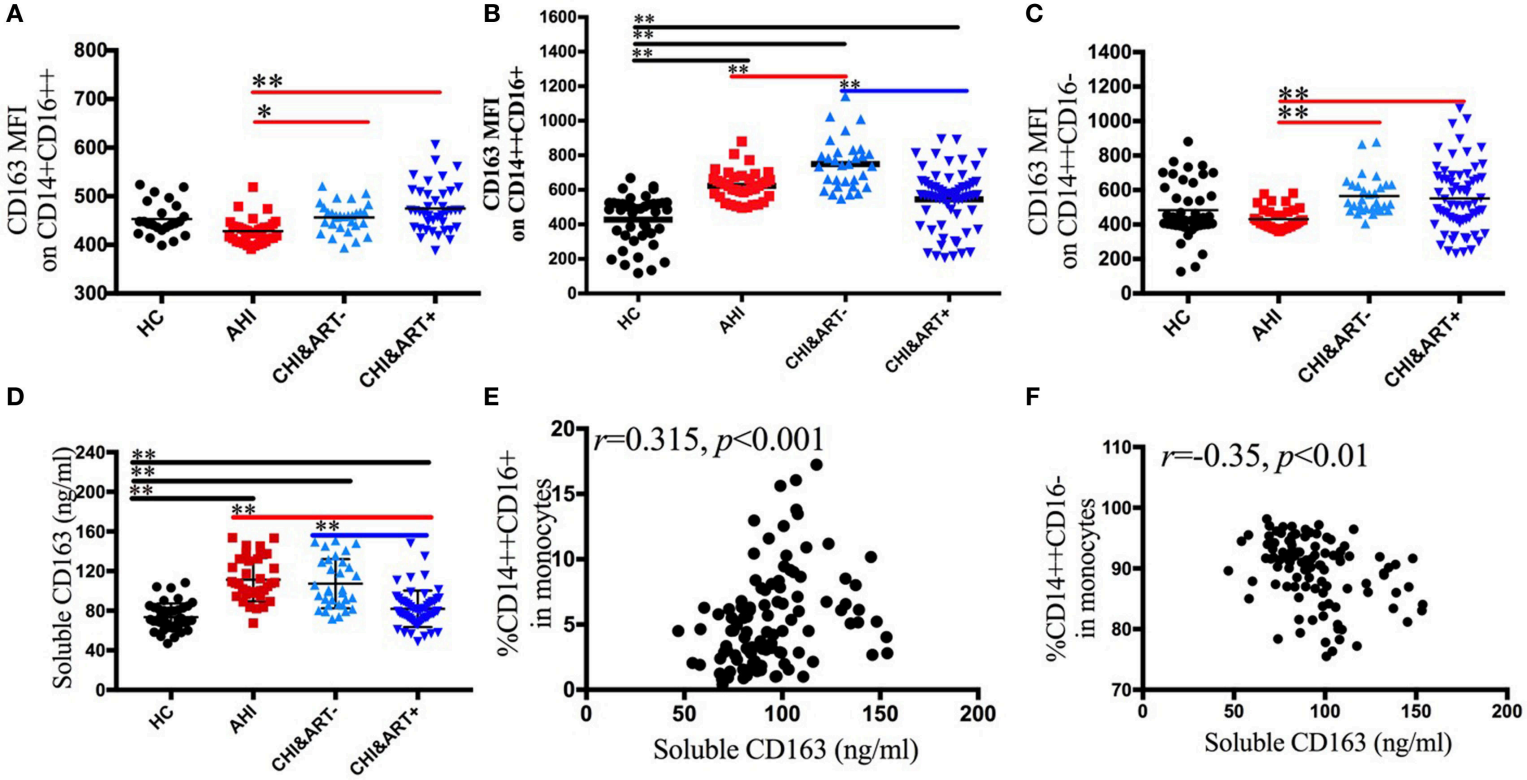
**Perturbations of surface CD163 on the three monocyte subsets and soluble CD163 in HIV-1-infected individuals**. The median fluorescence intensity of surface CD163 on the CD14+CD16++ **(A)**, CD14++CD16+ **(B)**, and CD14++CD16− **(C)** monocytes in healthy controls (HC), the patients with acute HIV-1 infection (AHI), chronic HIV-1-infected cART-naïve patients (CHI&ART−), and chronic HIV-1-infected patients with undetectable viral load after cART (CHI&ART+). The concentrations of plasma sCD163 **(D)** in HC, AHI, CHI&ART−, and CHI&ART+. All *p* values were calculated using an ANOVA, Student’s *t*-test, or the Mann–Whitney *U* test, *p < 0.05, **p < 0.01. Correlations between the concentrations of plasma sCD163 and the frequency of CD14++CD16+ monocytes **(E)**, as well as CD14++CD16− monocytes **(F)** calculated by the Spearman correlation test (HC, AHI, CHI&ART−, and CHI&ART+ groups).

To characterize the phenotypic changes of the three monocyte subsets, we analyzed the expression pattern of plasma sCD163 and the following surface markers: scavenger receptors CD163 (Figures 2A–C) and activation and maturation marker HLA-DR. Both the median fluorescence intensity (MFI) of surface CD163 on intermediate CD14++CD16+ monocytes and the concentrations of plasma sCD163 in AHI and CHI&ART− individuals are significantly higher than those in HC (Figures 2B,D). A significant decrease in plasma sCD163 levels and intermediate monocyte surface CD163 density is found in the chronic HIV-1-infected individuals after cART, but surface CD163 density on intermediate monocytes and sCD163 levels in CHI&ART+ still higher than those in HC (Figures 2B,D). In acute HIV-1-infected patients, surface CD163 density on intermediate monocytes is higher than those in HC but lower than those in chronic HIV-1-infected individuals without cART. The concentrations of plasma sCD163 are positively correlated with the frequency of intermediate CD14++CD16+ monocytes (Figure 2E), but inversely correlated with classical CD14++CD16− monocytes (Figure 2F).

**Figure 3:**
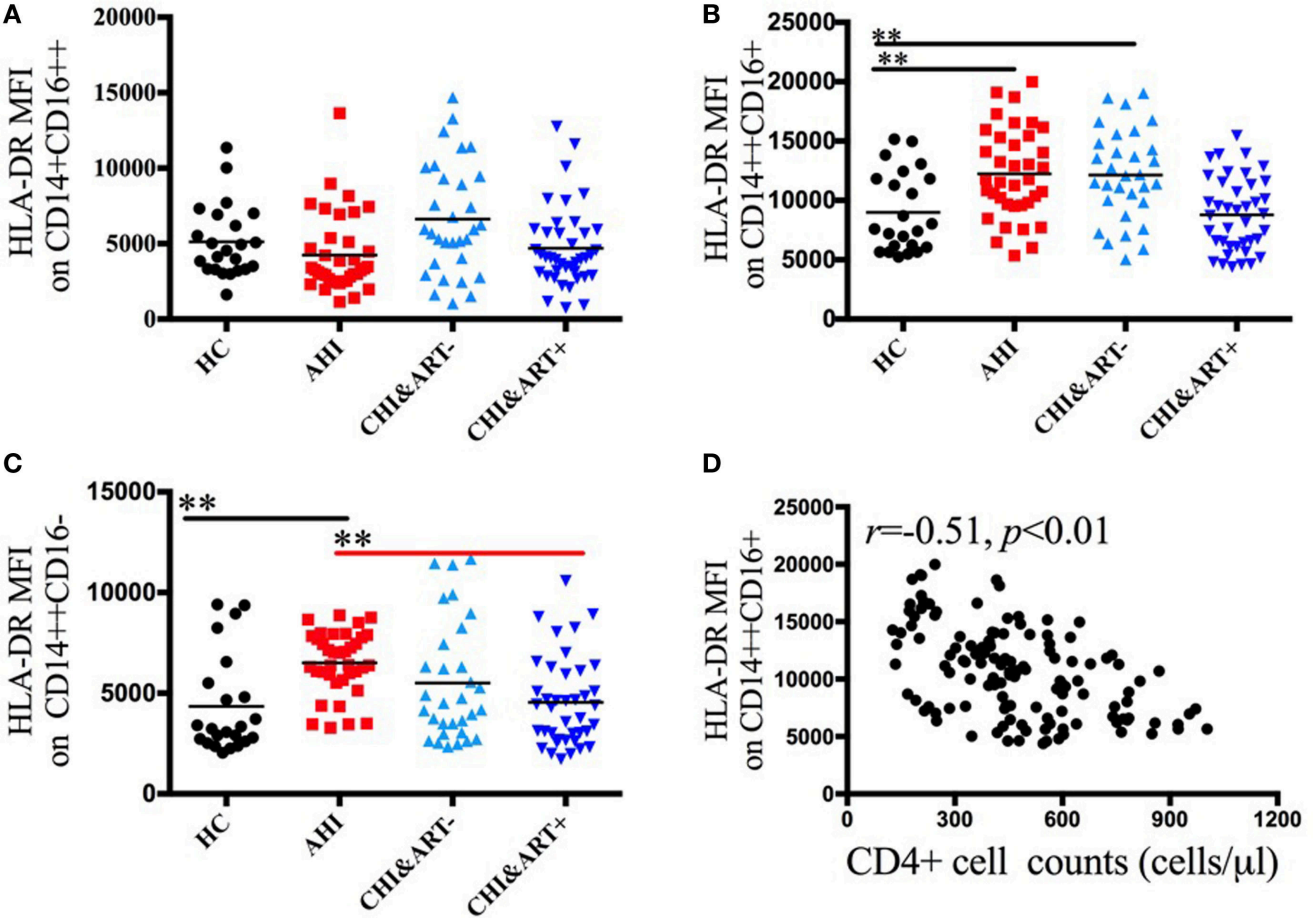
**Perturbations of surface HLA-DR on the three monocyte subsets in HIV-1-infected individuals**. The median fluorescence intensity (MFI) of surface HLA-DR on CD14+CD16++ **(A)**, CD14++CD16+ **(B)**, and CD14++CD16− **(C)** monocytes in healthy controls (HC), the patients with acute HIV-1 infection (AHI), chronic HIV-1-infected cART-naïve patients (CHI&ART−), and chronic HIV-1-infected patients with undetectable viral load after cART (CHI&ART+). All *p* values were calculated using an ANOVA, Student’s *t*-test, or the Mann–Whitney *U* test, *p < 0.05, **p < 0.01. Correlations between HLA-DR MFI on CD14++CD16+ monocytes and the CD4 cell counts **(D)** calculated by the Spearman correlation test (HC, AHI, CHI&ART−, and CHI&ART+ groups).

Figure 3 displays the dynamic changes of monocyte surface marker HLA-DR and its association with disease progression. The MFI of HLA-DR on intermediate CD14++CD16+ and classical CD14++CD16− monocytes in acute HIV-1-infected patients is significantly higher than that in HC (Figures 3B,C). The HLA-DR density on intermediate CD14++CD16+ monocytes is inversely correlated with the CD4+ cell counts (Figure 3D).

### The Association between Monocyte Subsets and IFN-γ, IL-4, IL-17, and TNF-α Producing CD4+ T Cells

**Figure 4:**
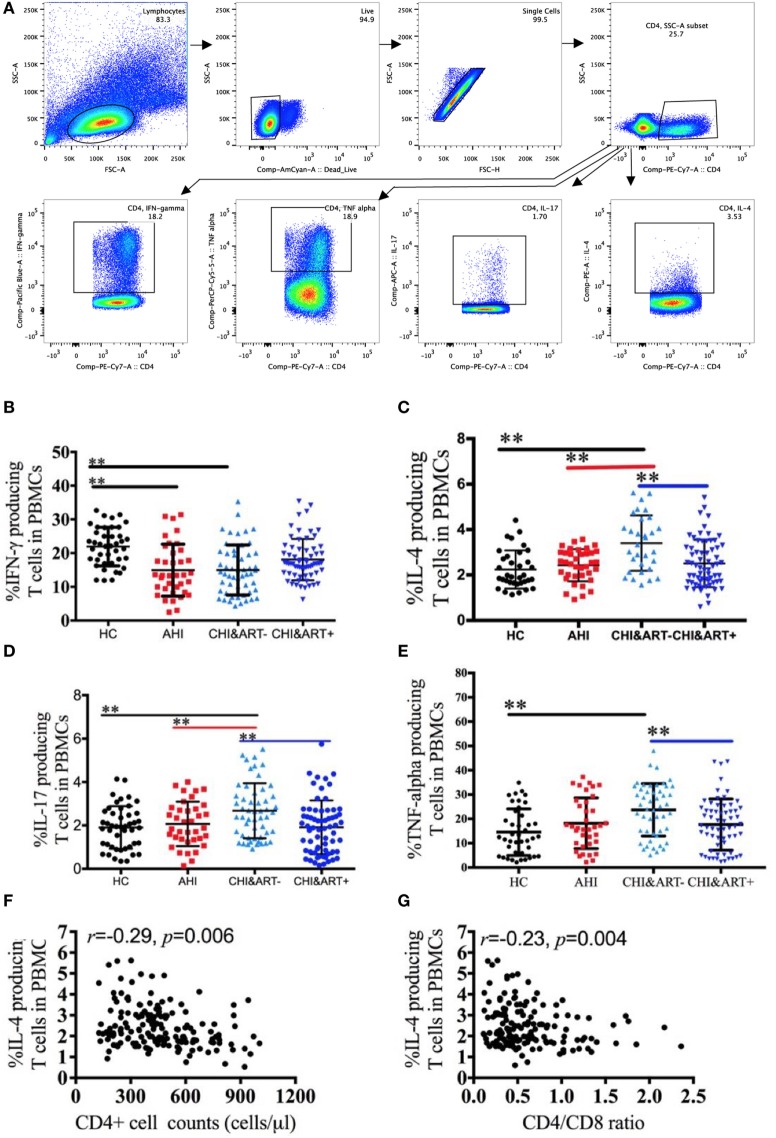
**Perturbations of IFN-γ, interleukin (IL)-4, IL-17, and TNF-α producing CD4+ T cells in HIV-1-infected individuals**. Intracellular IFN-γ, IL-4, IL-17, and TNF-α staining was performed using peripheral blood mononuclear cells (PBMCs) after stimulation with PMA, ionomycin, and brefeldin A for 5 h. The gating strategy is indicated **(A)**. Frequencies of IFN-γ **(B)**, IL-4 **(C)**, IL-17 **(D)**, and TNF-α **(E)** producing CD4+ T cells analyzed by flow cytometry in healthy controls (HC), chronic HIV-1-infected cART-naïve patients (CHI&ART−), and chronic HIV-1-infected patients with undetectable viral load after cART (CHI&ART+). All *p* values were calculated using an ANOVA, Student’s *t*-test, or the Mann–Whitney *U* test, *p < 0.05, **p < 0.01. Correlations between the frequencies of IL-4 producing CD4+ T cells and CD4+ T cell counts **(F)** as well as the CD4/CD8 ratio **(G)** calculated by the Spearman correlation test (HC, AHI, CHI&ART−, and CHI&ART+ groups).

The gating strategy of the IFN-γ, IL-4, IL-17, and TNF-α producing CD4+ T cells is shown in Figure 4A. Compared with HC, chronic HIV-1-infected individuals without cART have a lower frequency of IFN-γ producing CD4+ T cells (Figure 4B) but a higher frequency of IL-4 producing CD4+ T cells (Figure 4C). The frequency of IL-17 (Figure 4D) and TNF-α producing CD4+ T cells (Figure 4E) in chronic HIV-1-infected individuals without cART is higher than that in HC. The frequency of IL-4 producing CD4+ T cells is negatively associated with CD4+ T cell counts (Figure 4F) as well as with the CD4/CD8 ratio (Figure 4G).

**Figure 5:**
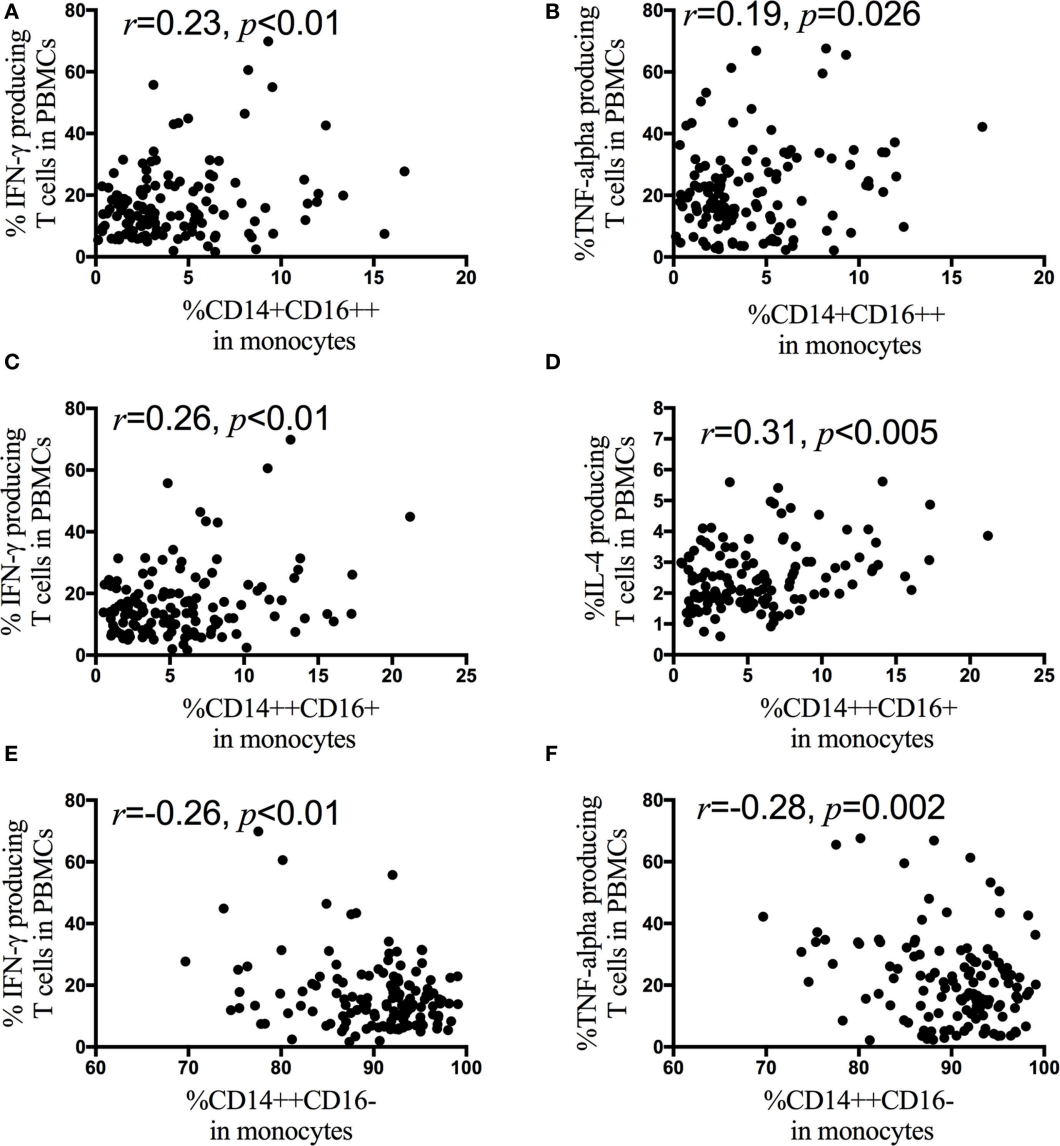
**Correlations between monocyte subsets and the frequencies of IFN-γ, interleukin (IL)-4, IL-17, and TNF-α producing CD4+ T cells in HIV-1-infected individuals intracellular IFN-γ, IL-4, IL-17, and TNF-α staining was performed using peripheral blood mononuclear cells (PBMCs) after stimulation with PMA, ionomycin, and brefeldin A for 5 h**. The frequency of the CD14+CD16++ monocytes is positively associated with the percentage of IFN-γ **(A)** and TNF-α **(B)** producing CD4+ T cells, the frequency of CD14++CD16+ monocyte subsets is positively associated with the frequency of IFN-γ **(C)** and IL-4 **(D)** producing CD4+ T cells in HIV-1-infected patients. The frequency of CD14++CD16− monocytes is negatively associated with the percentage of IFN-γ **(E)** and TNF-α **(F)** producing CD4+ T cells. The association is calculated by the Spearman correlation test (AHI, CHI&ART−, and CHI&ART+ groups).

Next, correlation analyses are performed between monocyte subsets and the IFN-γ, IL-4, IL-17, and TNF-α producing CD4+ T cells in HIV-1-infected patients. The frequency of the non-classical monocyte CD14+CD16++ subsets is positively associated with the percentage of IFN-γ (Figure 5A) and TNF-α (Figure 5B) producing CD4+ T cells, but the frequency of the classical monocyte (CD14++CD16−) subsets is negatively associated with the percentage of IFN-γ (Figure 5E) and TNF-α producing CD4+ T cells (Figure 5F). In contrast to the non-classical CD14+CD16++ and classical CD14++CD16− monocyte subsets, the frequency of the intermediate CD14++CD16+ monocyte subsets is positively associated with the frequency of IFN-γ (Figure 5C) and IL-4 (Figure 5D) producing CD4+ T cells. The associations between monocyte subsets and the IFN-γ, IL-4, IL-17, and TNF-α producing CD4+ T cells are further analyzed in AHI (Figure S1 in Supplementary Material), CHI&ART− (Figure S2 in Supplementary Material) and CHI&ART+ (Figure S3 in Supplementary Material) patients, separately. In AHI patients, the frequency of the intermediate CD14++CD16+ monocyte subsets is positively associated with the frequency of IL-4 (Figure S1D in Supplementary Material), whereas this subset is positively associated with the percentage of IFN-γ (Figure S2C in Supplementary Material) and IL-4 (Figure S2D in Supplementary Material) producing CD4+ T cells in CHI&ART− patients. However, in CHI&ART+ patients, there is no association between monocyte subsets and the IFN-γ, IL-4, IL-17, and TNF-α producing CD4+ T cells (Figure S3 in Supplementary Material).

## Discussion

In the study, we evaluated the dynamic changes of monocyte subsets and their surface markers in acute and chronic HIV-1-infected individuals, we also analyzed the association between monocyte subsets and Th cell differentiation. We found that in the acute HIV-1-infected individuals, the frequency of the intermediate CD14++CD16+ monocyte subsets, the HLA-DR density and CD163 density on CD14++CD16+ monocytes, and plasma CD163 were significantly higher than those in HC, but lower than those in chronic HIV-1-infected individuals. Intermediate CD14++CD16+ monocytes and their HLA-DR expression on intermediate CD14++CD16+ monocytes were inversely correlated with the CD4 cell counts, and the intermediate CD14++CD16+ monocytes were positively correlated with plasma sCD163. The frequency of IL-4 producing CD4+ T cells is negatively associated with CD4 cell counts as well as with the CD4/CD8 ratio. In contrast to non-classical CD14+CD16++ and classical CD14++CD16− monocyte subsets, the frequency of the intermediate CD14++CD16+ monocytes was positively associated with the frequency of IFN-γ and IL-4 producing CD4+ T cells in HIV-1-infected patients. The intermediate CD14++CD16+ monocyte subsets were positively associated with IFN-γ (CHI&ART− group) and IL-4 (AHI and CHI&ART− groups) producing CD4+ T cells. There was no association between the monocyte subsets and T helper cell responses in CHI&ART+. Intermediate monocyte subsets, surface CD163 density on intermediate monocytes, and plasma sCD163 levels in CHI&ART+ still higher than those in healthy control, abnormalities of the three monocyte subsets still existed despite suppression of HIV after cART.

Our findings indicated that monocytes were heterogeneous with subset-specific phenotypes and functions during HIV-1 infection, intermediate CD14++CD16+ monocytes were correlated with disease progression in acute and chronic HIV-1-infected individuals, and the three monocyte subsets played different roles in Th cell differentiation in HIV-1-infected patients. The abnormalities of three monocyte subsets may be related to chronic immune activation despite suppression of HIV after cART.

Based on the expression of the CD14 and CD16 antigen, human blood monocytes were initially classified into two subsets: CD14+CD16− and CD14+CD16+ monocyte subsets (17). Recently, monocytes have been subdivided into three subsets by official nomenclature, each subset displays different cytokine profiles and immune functions. Classical monocytes CD14++CD16− play crucial roles in defending against microbial pathogens, intermediate CD14++CD16+ monocytes are associated with antigen presentation and inflammatory, and non-classical CD14+CD16++ monocytes exhibit patrolling properties (18). A strong increase of intermediate and a concomitant decrease of classical monocytes has been found in patients with rheumatoid arthritis (19). Similar to rheumatoid arthritis patients, in this study, an expansion of intermediate monocytes and a reduction of classical monocytes were found in acute and chronic HIV-1-infection. cART produces a profound suppression of HIV replication but fails to eliminate chronic immune activation completely, and the persistent hyperactivity is associated with the emergence of non-AIDS morbidity (20). Perturbations of the three monocyte subsets were found in this study despite the suppression of HIV after cART, which may be responsible for chronic immune activation. The expansion of the intermediate monocytes may be due to an enhanced survival upon HIV-1 infection (21) and the upregulation of surface CD16 on the classical subsets (22).

Burdo et al. showed that sCD163 levels were positively correlated with CD14+CD16+ monocytes (23). CD14+CD16+ monocytes were further subdivided into CD14++CD16+ and CD14+CD16++ subsets. We found that plasma sCD163 was only positively correlated with the intermediate CD14++CD16+ subsets, and there was no association between plasma sCD163 and the non-classical CD14++CD16+ subsets. HIV-1-infected individuals after cART had higher levels of plasma sCD163 than those of HC, which indicated an important role of activated monocytes in HIV-1-related chronic immune activation. In the study, we found that the MFI of HLA-DR on intermediate monocytes in acute HIV-1-infected patients was significantly higher than that in HC, which implied that intermediate monocytes have a crucial role in regulating the adaptive immune responses in acute HIV-1 infection.

Monocytes have many immunological functions including antigen presentation, which makes them a link between the innate and adaptive immune systems. Th1, Th2, and Th17 CD4+ T cells are characterized exclusively by differences in cytokine expression: Th1 cells produce IL-2, IL-12, and interferon IFN-γ, Th2 cells express IL-4, IL-5, and IL-13, and Th17 cells produce IL-17, IL-17F, and IL-22. The dynamic changes of Th1, Th2, and Th17 in HIV-1-infected individuals have been reported by several groups: a reduced percentage of IFN-γ (Th1) producing cells and an increased percentage of IL-4 (Th2) producing cells (24) as well as conflicting results regarding IL-17 producing cells (25, 26). Human peripheral monocytes have been subdivided into three subsets with specific functions. In this study, we found that the percentages of IFN-γ and TNF-α producing CD4+ T cells were positively associated with the non-classical CD14+CD16++ monocyte subsets but were negatively associated with the percentages of the classical monocyte CD14++CD16− subsets. Compared with CD4 T cells co-cultured with CD16-derived dendritic cells (MoDC), CD4 T cells co-cultured with CD16+ MoDC secreted more IL-4 (27). Here, we showed an increased frequency of IL4 producing CD4+ T cells in HIV-1-infected individuals, and the frequency of IL4 producing CD4+ T cells was negatively associated with CD4 cell counts as well as with the CD4/CD8 ratio. The intermediate CD14++CD16+ monocyte subsets were positively associated with IFN-γ (CHI&ART− group) and IL-4 (AHI and CHI&ART− groups) producing CD4+ T cells. cART lead to virologic suppression and reduction of persistent immune activation, despite the abnormalities of the three monocyte subsets in individuals on cART, the associations between the monocyte subsets and the IFN-γ, IL-4, IL-17, and TNF-α producing CD4+ T cells have not been found in HIV-1-infected patients on cART with undetectable viral loads. Monocytes are precursors for DCs and macrophages, and blood monocytes can replenish specialized macrophages and DCs on demand (28). Compared with HIV-1-treated monocytes, HIV-1-treated MoDCs have different effects on IL17+CD4+ T cells (29). An *in vitro* PBMC stimulation culture system was used in this study, in which PBMCs were stimulated with PMA/ionomycin. Direct contact of monocytes and T cells is important for T cell differentiation (30), and the monocytes and T cell co-culture system will be used in future studies to better understand the roles of monocytes in Th cell differentiation.

In summary, we evaluated the perturbations of monocyte subsets and their association with Th cell differentiation in acute and chronic HIV-1-infected individuals. Our observations provide new insight into the roles of monocyte subsets in HIV pathogenesis, particularly during AHI, and our findings may be helpful for the treatment of HIV-related immune activation.

## Author Contributions

BS, LL, and HW conceived the study, designed the experiments, and analyzed the data. PC, XZ, HX, and GZ performed the experiments; BS, TZ, WX, LD, LS, and YF contributed to reagents and materials; and PC, BS, LL, and HW wrote the article. All authors read and approved the final manuscript.

## Conflict of Interest Statement

The authors declare that the research was conducted in the absence of any commercial or financial relationships that could be construed as a potential conflict of interest.
