# Supplementary figures and images for: Perturbations of Monocyte Subsets and Their Association with T Helper Cell Differentiation in Acute and Chronic HIV-1-Infected Patients
Source: Front Immunol. 2017 Mar 13;8:272. doi: 10.3389/fimmu.2017.00272 (PMC5347116; doi:10.3389/fimmu.2017.00272)

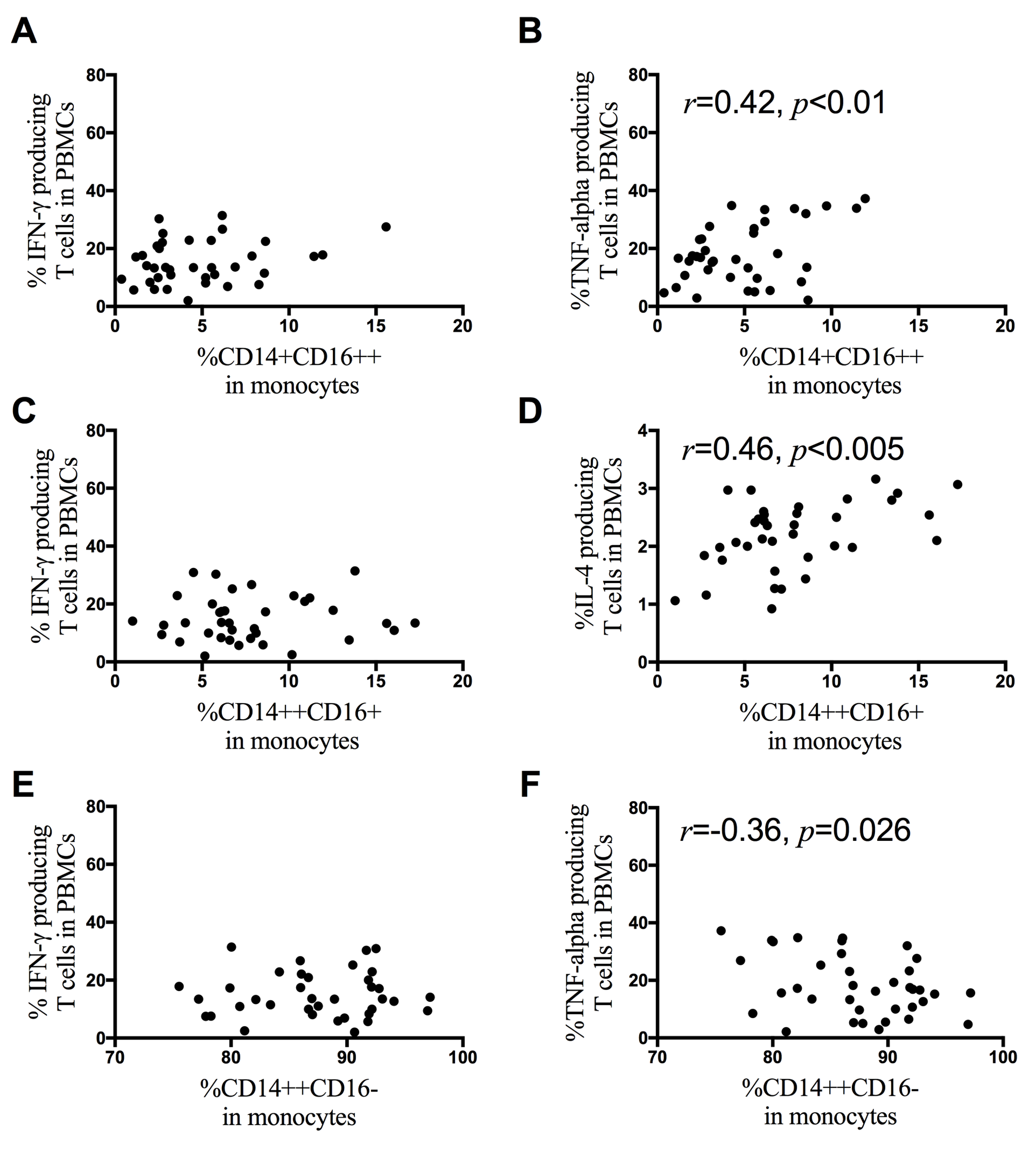

Supplement: Supplementary file 1 [file Image_1.TIF]

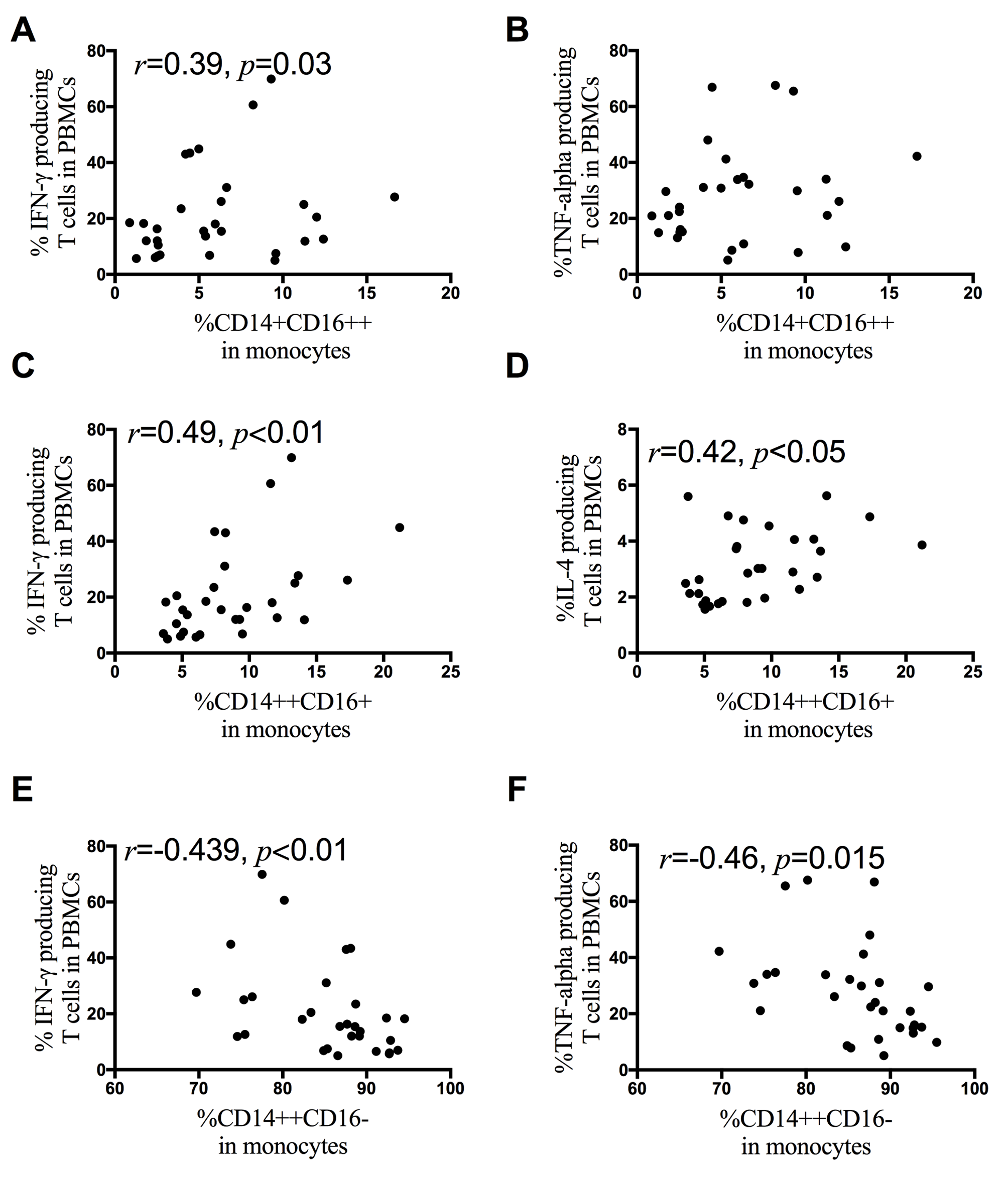

Supplement: Supplementary file 2 [file Image_2.TIF]

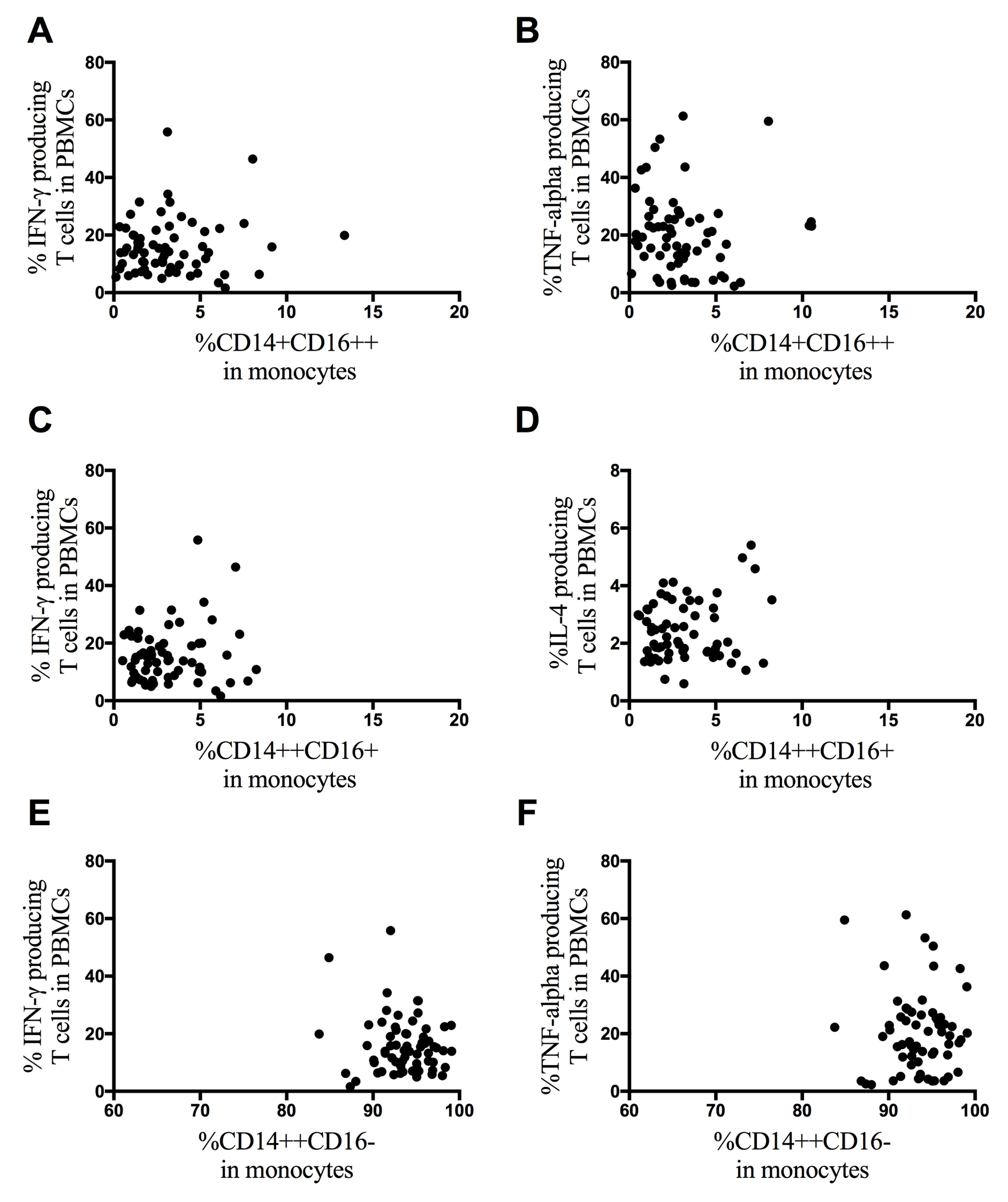

Supplement: Supplementary file 3 [file Image_3.TIF]
